# Geographical disparities in risks of emergency department visits due to intentional self-harm in Florida: a retrospective ecological study

**DOI:** 10.7717/peerj.21963

**Published:** 2026-11-12

**Authors:** Howard Onyuth, Agricola Odoi

**Affiliations:** Biomedical and Diagnostic Sciences, University of Tennessee, Knoxville, TN, United States of America

**Keywords:** Intentional self-harm, Emergency department visits, Geographic disparities, GIS, Geographical information systems, Spatial epidemiology, Tango’s spatial scan statistics, Spatial cluster detection, Florida

## Abstract

**Background:**

Intentional self-harm is a major and growing public health concern in Florida, contributing to thousands of emergency department (ED) visits each year. Although there is evidence that the burden of intentional self-harm varies by geographical location, little is known about disparities of intentional self-harm related ED visit risks in Florida. Understanding these disparities is important for guiding health planning, allocation of resources, and tailoring efforts to mitigate the problem. Therefore, the objectives of this study were to investigate county-level geographic disparities in intentional self-harm-related ED visit risks in Florida.

**Methods:**

Retrospective ED data were obtained from Florida Health Charts, a publicly available data dashboard maintained by the Florida Department of Health. County-level age-adjusted intentional self-harm-related ED visit risks were calculated for four time periods: 2016–2017, 2018–2019, 2020–2021 and 2022–2023. Tango’s spatial scan statistics were used to identify high-risk clusters. Two sample tests of proportions were used to compare proportions of counties and populations in high-risk clusters between rural and urban areas.

**Results:**

Age-adjusted intentional self-harm-related ED visit risks decreased from 139.4 to 127.4 visits per 100,000 persons over the study period. Counties in the northwest, northeast, central, and southwest Florida tended to have high risks. Statistically significant high-risk clusters of intentional self-harm ED visits were detected in multiple predominantly rural areas, with seven clusters in 2016–2017 (relative risks (RR) = 1.2–2.2), nine clusters in 2018–2019 (RR = 1.2–2.8), five clusters in 2020–2021 (RR = 1.3–1.9), and six clusters in 2022–2023 (RR = 1.2–1.9). The proportion of counties in high-risk clusters increased from 46.3% in 2020–2021 to 61.2% in 2022–2023. While there were no significant (*p* > 0.05) differences between rural and urban counties in earlier periods (2016–2021), by 2022–2023 significantly (*p* = 0.001) more rural (81.3%) than urban (42.9%) counties were part of high-risk clusters. Over the study period, 34.3% of the counties were persistently part of high-risk clusters. There was a significantly (*p* < 0.0001) higher proportion of rural than urban residents living in high-risk clusters; increasing from 57.2% in 2016–2017 to 71.9% in 2022–2023 in rural areas compared to an increase from 35.0% in 2016–2017 to 41.8% in 2022–2023 in urban areas.

**Conclusion:**

Despite the declining trend, geographic disparities of intentional self-harm related ED visit risks exist in Florida. Study findings may be useful for informing future studies, targeted intentional self-harm prevention strategies, and need-based resource allocation geared at reducing disparities and improving population health for all.

## Introduction

Intentional self-harm, defined as the deliberate destruction of one’s own body tissue with or without suicidal intent, is a growing public health problem contributing to millions of emergency department (ED) visits each year (Prichett et al., 2025). Approximately 5.37 million ED visits reported in the US between 2019 and 2020 were due to intentional self-harm (Weiss & Jiang, 2021; Bommersbach, Olfson & Rhee, 2024). Individuals with a history of intentional self-harm have 30 times higher suicide risk than the general population (Bommersbach, Olfson & Rhee, 2024; Prichett et al., 2025), with 39% of those who committed suicide having visited the ED for intentional self-harm at least once in the year preceding their death (Ting et al., 2012). However, the statistics and impacts of intentional self-harm are underestimated due to incomplete surveillance data and underreporting (Ross et al., 2023; Kraus & Ferry, 2024). Consequently, much of the existing evidence relies on ED data, which captures only a subset of self-harm events and reflects patterns of healthcare utilization and service availability rather than underlying burden of self-harm (Bommersbach, Olfson & Rhee, 2024; Sandahl et al., 2024).

The economic burden associated with intentional self-harm ED visits in the US is estimated at $26 billion annually (Bommersbach, Olfson & Rhee, 2024), with an estimated 5.32 billion attributed to ED treat and release visits alone (Centers for Disease Control and Prevention, 2023). The US states in the top quantile based on per patient cost of intentional self-harm are concentrated in the Great Lakes, Southeast, Mideast, and New England (Rockett et al., 2023). The per patient median ED cost of intentional self-harm injury in Florida, a Southeast state, is estimated at $674 (Peterson, Haileyesus & Stone, 2024).

Florida, like many states in the US, struggles with high numbers of intentional self-harm ED visits. For instance, in 2023, there were 12,163 intentional self-harm ED visits in the state (Florida Department of Health, 2026). In addition, Florida has a highly diverse population, with pronounced rural–urban socio-economic disparities that influence mental health outcomes, availability of mental health services, and access to care (Bigham et al., 2023). This potentially leads to higher rates of intentional self-harm ED visits in certain communities. For example, as of 2022, 47.2% of the Miami-Dade population was living below the federal poverty level and 90.4% of its mental health patients were not receiving the mental health care they needed (Florida Department of Health, 2022).

There is evidence that the burden of intentional self-harm ED visits varies by geographical location (Rockett et al., 2023). However, past studies have investigated these at the national or state spatial scales, (Ting et al., 2012; Sullivan & Camargo Jr, 2015; Bommersbach, Olfson & Rhee, 2024; Litenski et al., 2024), which can obscure sub-state variation, mask local challenges, and limit need-based efforts to address the problem at the local level (Onyuth et al., 2025). Understanding disparities in intentional self-harm ED visits at the county level is important because counties serve as primary administrative units for implementation of public health programs and points of data-driven intervention for at risk local communities (Florida Department of Health, 2020; Florida Department of Health, 2023). Counties also coordinate with state agencies, EDs, and behavioral health providers to facilitate access to mental health services, often operating or funding public mental health clinics, crisis stabilization units, and community outreach programs that serve as safety nets for high-risk, low income, and indigent populations (Florida Department of Health, 2023). Therefore, identification of disparities in intentional self-harm ED visits at the county-level may help guide local health planning and tailor efforts to mitigate the problem, that would not be apparent from state or national spatial analyses (Florida Department of Health, 2022). Unfortunately, information on geographic disparities of intentional self-harm ED visits in Florida is scarce, and yet this information is useful for guiding need-based resource allocation intended to curb the problem (Bommersbach, Olfson & Rhee, 2024). Therefore, the objective of this study was to investigate county-level geographic disparities in intentional self-harm-related ED visit risks in Florida.

## Materials and Methods

### Ethics review

This study was reviewed by the University of Tennessee Institutional Review Board (IRB) (IRB Number: UTK IRB-25-09098-XM) and determined to be exempt under 45 CFR 46.101. Category 4i: Secondary research using publicly available data. The analysis used publicly available data aggregated at the county level with no individual level or identifiable information.

### Study area

This study was conducted in Florida, a Southeast state of the United States of America, composed of 67 counties. Florida has a population of 21.5 million people, of which 51.5% are White (non-Hispanic/Latino), 26.5% Hispanic or Latino, 15.1% Black or African American, 3.0% Asian, and 3.9% other races (United States Census Bureau, 2020). The state includes a mix of urban and rural counties (Fig. 1). Rural–urban classification of counties was based on the US Department of Commerce 2020 Census (United States Department of Commerce, 2020). Based on this classification, a county is considered rural if it has a population of 75,000 or fewer, or a population of 125,000 or fewer that is contiguous to a county with 75,000 or fewer residents; or any municipality located within a county meeting either of these criteria. Counties that do not meet the above criteria were considered urban counties (United States Department of Commerce, 2020).

**Figure 1:**
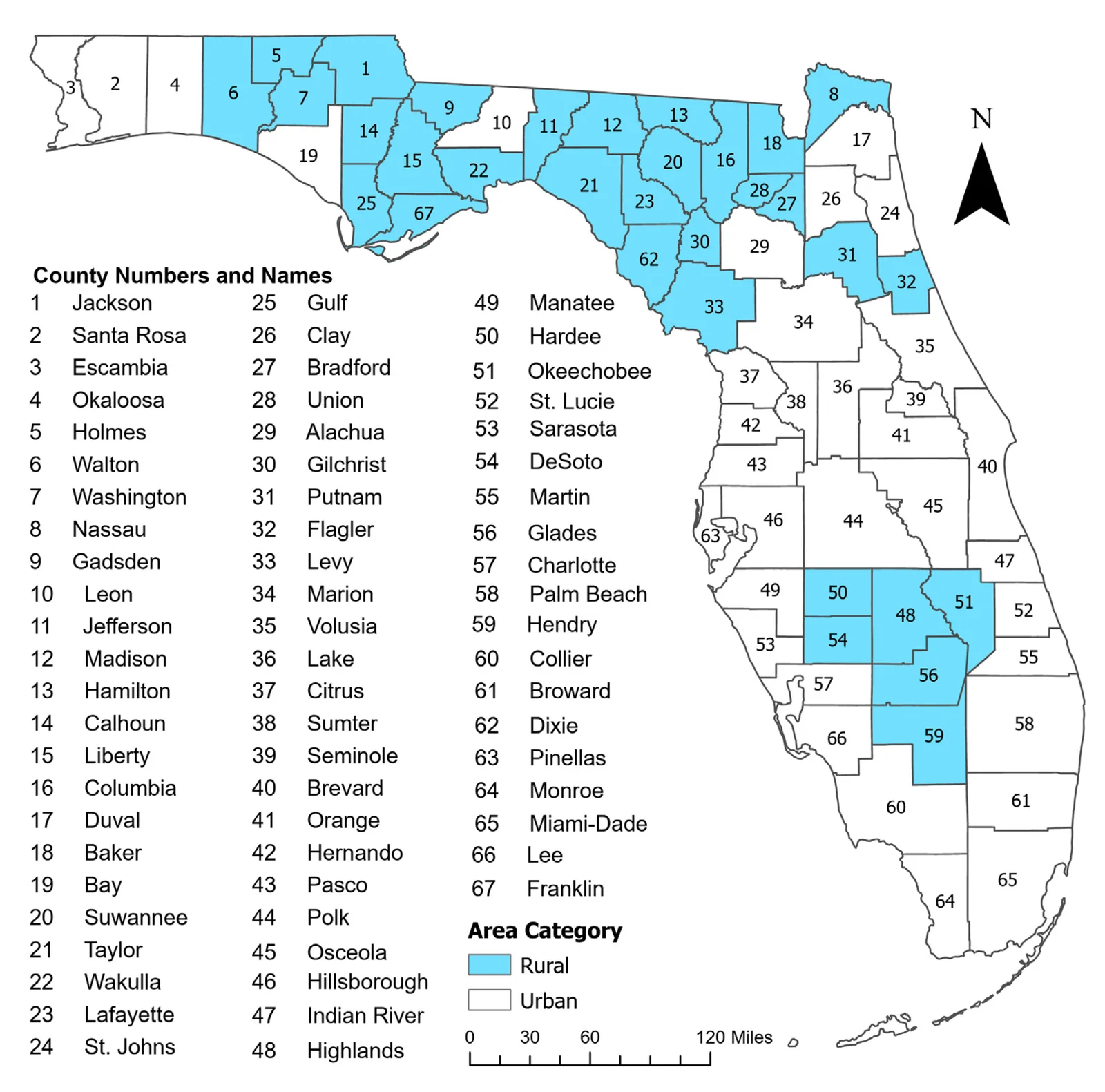
Map of Florida showing county names and the urban/rural classification of the counties. The map was created by the authors. The cartographic boundary file used to create the map was obtained from the United States Census Bureau TIGER Geodatabase. Rural–urban classification was based on the US Department of Commerce 2020 Census classification.

Miami-Dade is the most urbanized county, with approximately 2.7 million residents (Florida Department of Health, 2022). In contrast, Liberty County is the most rural, with only about 7,974 residents (United States Census Bureau, 2020). Collier County, an urban county, is the largest county by land area covering 1,996.9 square miles, while Union County, a rural county, is the smallest spanning only 243.6 square miles (United States Census Bureau, 2010).

### Data sources

Intentional self-harm Emergency Department (ED) data were obtained from Florida Health Charts, a publicly available data dashboard maintained by the Florida Department of Health (Florida Department of Health, 2024). Only ED visits with ICD-10-CM codes explicitly indicating intentional self-harm were included. Specifically, for T36–T50 and T51–T65 codes, the Florida department of Health restricted inclusion to records where the 5th or 6th character indicated intentional self-harm (for example, character value = “2”), consistent with ICD-10-CM coding conventions for injury intent. Thus, records for ED visits due to intentional self-harm where the ICD-10 codes were listed as the principal diagnosis were identified by the Florida Department of Health. The ED visits were classified by the Florida Department of Health in cases where the principal diagnosis was any ICD-10 injury code (*i.e.,* S00-S99, T07-T34, T36.9/T37.9/T39.9/T41.4/T42.7/T43.9/T45.9/T47.9/T49.9 with 5th character of 1-4, T36-T50 with 6th character of 1-4, T51-T76, T79, O9A.2-O9A.5, T84.04, M97; 7th character of A, B, C, or blank). Intentional self-harm ICD-10-CM codes included: T14.91, T36–T50 with 6th character of 2, T36.9/T37.9/T39.9/T41.4/T42.7/T43.9/T45.9/T47.9/T49.9 with 5th character of 2, and T51–T65 with 6th character of 2, T51.9/T52.9/T53.9/T54.9/T56.9/T57.9/T58.0/T58.1/T58.9/ T59.9/T60.9 /T61.0/T61.1/T61.9/T62.9/T63.9/T64.0/T64.8/T65.9 with 5th character of 2, T71 with 6th character of 2, X71-X83 as any diagnosis or external cause of injury. The data included multiple ED visits per individual. The dataset used did not have unique identifier per patient and so it was not possible to determine frequency of unique individuals. Therefore, each record represents a self-harm visit, not a unique individual since an individual can visit more than once.

County level population estimates were obtained from the 2016, 2018, 2020, and 2022 American community survey and were used as denominators for the 2016–2017, 2018–2019, 2020–2021 and 2022–2023 time periods, respectively (American Community Survey, 2025). Rural–urban classification data for each county were obtained from the US Department of Commerce 2020 Census urban and rural areas (county and cities profiles (PL 94-171)) (United States Department of Commerce, 2020). County-level data on all ED visits in Florida for the years 2016–2023 were obtained from Florida Health Charts (Florida Department of Health, 2024). The 2023 geographic boundary files, used for cartographic displays, were obtained from the United States Census Bureau TIGER Geodatabase (United States Census Bureau, 2026). The datasets did not contain any missing values.

### Calculation of ED visits attributable to intentional self-harm

To contextualize variation in overall ED utilization across counties and time periods (2016–2017, 2018–2019, 2020–2021 and 2022–2023), the percentage of ED visits attributable to intentional self-harm for each county was calculated as the number of ED visits due to intentional self-harm divided by the total number of ED visits in that county over the same time period.

### Calculation of age-standardized ED visit risks, and cartographic displays

County-level data on emergency department visits due to intentional self-harm for each of the four time periods (2016–2017, 2018–2019, 2020–2021 and 2022–2023) were aggregated by age categories: <18 years, 18–34 years, 35–44 years, 45–64 years, and ≥65 years. The use of four two-year time periods was determined a priori to improve the stability and reliability of county-level estimates, particularly for counties with low populations or ED visit counts. Estimates of risk of ED visits due to intentional self-harm were calculated and directly age standardized to the 2010 US standard population using the “dstdize” command in STATA version 18 (Mehmetoglu & Jakobsen, 2022).

The percentage ED visits attributable to intentional self-harm and age-standardized risk of ED visits due to intentional self-harm were displayed as choropleth maps for each time period. The critical intervals used in the choropleth maps were first determined using Jenk’s optimization classification scheme for the time period which had the largest data range (2018–2019). For consistency and to enable comparison across time periods, the intervals used to display the 2018-2019 risks maps were also used in the choropleth maps for the 2016–2017, 2020–2021 and 2022–2023 risk maps.

### Cluster detection and identification

Tango’s flexible spatial scan statistics (FSSS), implemented in FleXScan version 3.1.2 (Tango & Takahashi, 2005), was used to identify both circular and irregularly shaped high-risk clusters of ED visit risks due to intentional self-harm for each of the four time periods (2016–2017, 2018–2019, 2020–2021 and 2022–2023). Under the null hypothesis of no spatial clustering, the case counts in each region (counties in this study) in the study area follows a Poisson distribution with mean proportional to the population at risk (Tango, 2021). Adjustment for age, a confounder, was performed by calculating age-adjusted counts (computed as age-adjusted risk of ED visits due to intentional self-harm*population at risk). Data preparation for cluster identification involved several steps: (1) preparation of the coordinate file, which contained coordinates (*i.e.,* latitudes and longitudes) of the geographic centroids of the spatial units of analysis (*i.e.,* counties); (2) creating queen first order contiguity spatial weights, a matrix definition file which showed the connectedness of an individual county to its neighbors; (3) creating a case file which contained data on the age-adjusted number of cases and population at risk in each county in the study area. The FSSS method systematically evaluates a large set of flexibly shaped scanning windows of varying sizes, using an adjacency matrix design, beginning with individual regions and expanding to include all combinations of contiguous neighboring regions up to a specified maximum size (Tango & Takahashi, 2012; Tango, 2021). For each potential cluster, a log-likelihood ratio (LLR) is calculated by comparing the observed and expected (if cases were randomly distributed) number of cases within the window (Tango, 2021). The cluster with the highest LLR is then identified as the primary cluster while additional non-overlapping clusters with lower LLR values are considered secondary clusters (Tango & Takahashi, 2012; Tango, 2021). The model inputs were case file, the coordinate file, and matrix definition file. Model specifications were as follows. Poisson model with a maximum scanning window size set to 15 to prevent detection of excessively large clusters (Tango, 2021). A restricted log-likelihood ratio (LLR) test with a default alpha of 0.2 was used to ensure only regions with elevated risks were included in high-risk clusters (Tango & Takahashi, 2012; Tango, 2021). Significance testing was assessed using 999 Monte Carlo permutations and a critical *p*-value threshold of 0.05 (Tango & Takahashi, 2012). Only clusters with a risk ratio ≥1.20 were reported to ensure counties with minimally elevated risks were not included (Takahashi, Yokoyama & Tango, 2010; Tango & Takahashi, 2012).

All geographic information system (GIS) manipulations and cartographic displays including mapping of high-risk clusters were performed in ArcGIS Pro (Environmental Systems Research Institute, 2024). To assess if the geographic distribution of the clusters changed from the beginning of the study period (2016–2017) to the end (2022–2023), visual comparisons of the maps were performed and high-risk clusters that persisted throughout the study period were identified and mapped using ArcGIS Pro (Environmental Systems Research Institute, 2024). A two-sample test of proportions was used to make rural–urban comparisons of counties, relative risks and populations in high-risk clusters.

## Results

### Temporal and geographical distribution of ED visit risks

#### Emergency department (ED) visits attributable to intentional self-harm

Of the 67.3 million ED visits reported in Florida during the study period, 0.15% (101,080/67,321,861) were attributable to intentional self-harm. The share of ED visits due to intentional self-harm varied geographically, with higher percentages observed in some counties in the southwest, northwest, northeast, and southern regions (Fig. 2). The highest percentages were observed in the northeastern rural county of Union (0.58% in 2016–2017, 0.57% in 2018–2019, 0.4% in 2020-2021), and the southern rural county of Glades (0.23% in 2022–2023). The rural counties that had the highest share of all ED visits attributable to intentional self-harm were surrounded by urban counties with similarly high percentages. Considering the whole study period, only three urban counties (Brevard, Hernando, and Pasco) had high percentages of ED visits attributable to intentional self-harm although they were not adjacent to rural counties.

**Figure 2:**
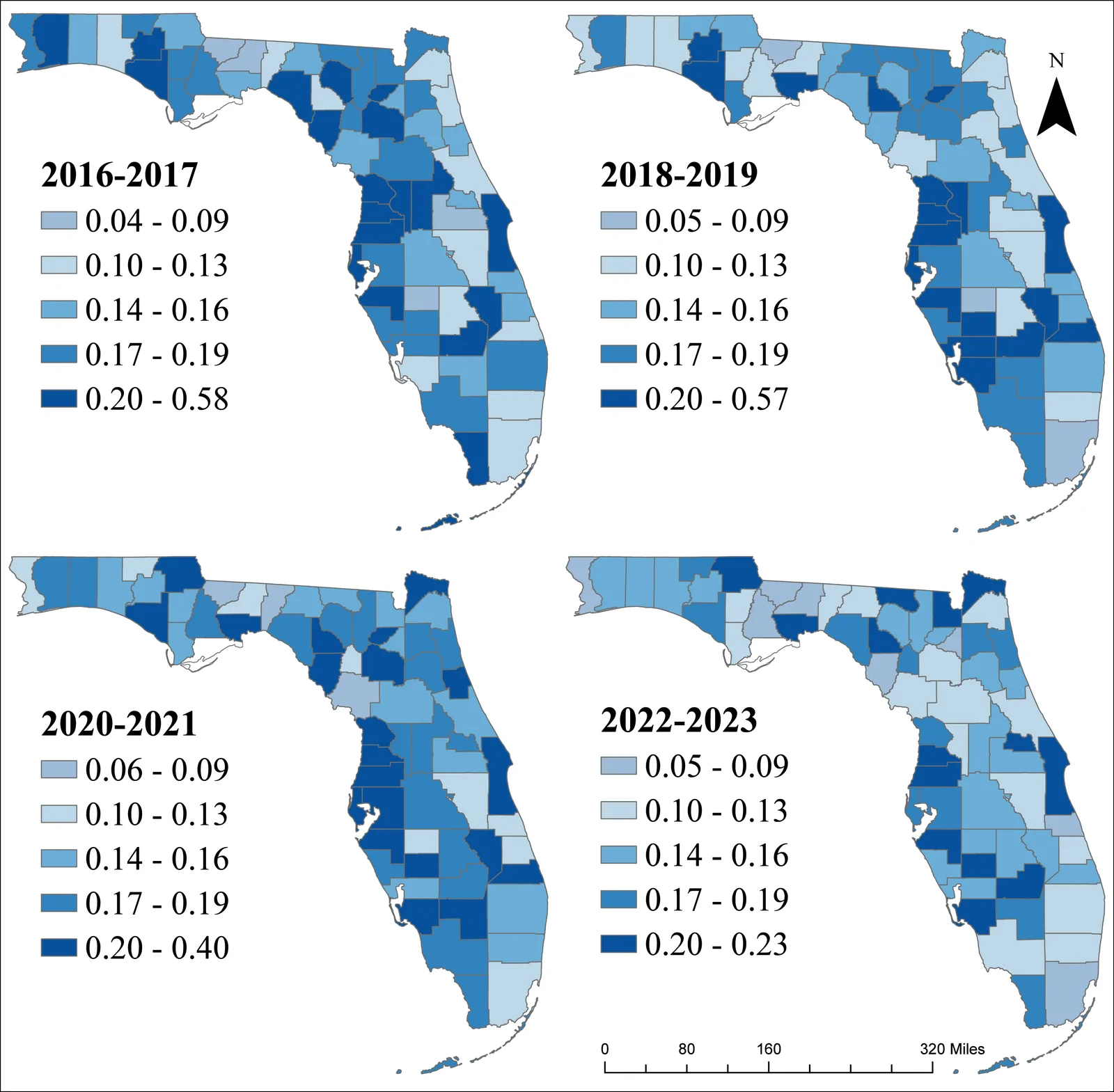
Percentage of emergency department visits attributable to intentional self-harm in Florida, 2016–2023. The map was created by the authors using ArcGISPro software. The cartographic boundary files were obtained from the United States Census Bureau TIGER Geodatabase. Data displayed were obtained from the Florida Department of Health.

#### Intentional self-harm ED visit risks

There was a slight decrease in the overall state age-adjusted risk of ED visits due to intentional self-harm from 139.4 visits per 100,000 persons in 2016–2017, to 133.4 in 2018–2019, 128.2 in 2020–2021, and 127.4 in 2022–2023. The risk of ED visits due to intentional self-harm varied geographically, with the northwestern, northeastern, central, and southwestern counties having high ED visit risks during each of the four time periods assessed (Fig. 3).

**Figure 3:**
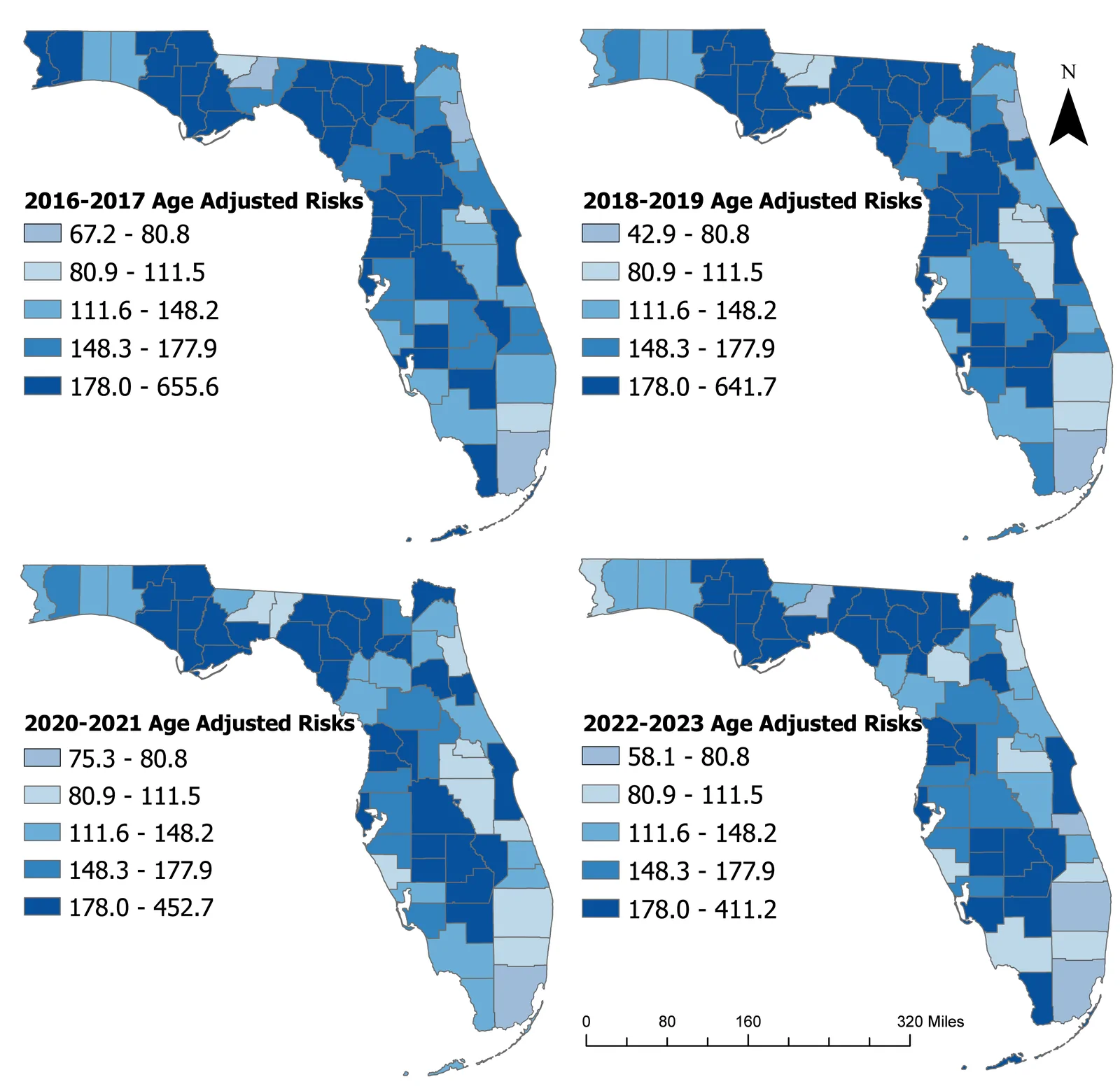
Age-adjusted risk of emergency department visits due to intentional self-harm in Florida, 2016–2023. The map was created by the author using ArcGISPro software. The cartographic boundary files were obtained from the United States Census Bureau TIGER Geodatabase. Data displayed were obtained from the Florida Department of Health.

The highest ED visit risks were observed in the northeastern rural counties of Union (655.6 per 100,000 persons in 2016-2017, to 641.7 in 2018–2019, and then 452.7 in 2020–2021) (Figs. 1 and 3). This high ED visit risk area was surrounded by other rural high ED visit risk counties in the northeastern region (*i.e.,* Columbia, Baker, Bradford, Madison, Taylor, Lafayette, Dixie, Suwannee, Hamilton, *etc*.). Other high ED visit risk rural counties were in the northwest (*i.e.,* Holmes, Washington, Jackson, Calhoun, Gulf, *etc*.), and southeastern (Okeechobee) parts of the state.

High ED visit risks were also observed in a few urban counties during the first three time periods: Escambia and Santa Rosa in the northwest, Pasco in the southwest, Lake and Marion in central Florida. These counties did not have high risks during the 2022–2023 period although they were surrounded by counties that had high risks of ED visits.

### Spatial clusters of high ED visit risks due to intentional self-harm

#### Geographic distribution and relative risks

Significant clusters of high ED visit risks due to intentional self-harm were observed in parts of the northwest, northeast, southwest, central, south, and southwest parts of Florida (Fig. 4). During 2016–2017, seven statistically significant clusters were identified, with relative risks (RRs) ranging from 1.2 to 2.2 (Table 1 and Fig. 4). The cluster with the highest ED visit risk (Cluster 6; RR = 2.2) was confined to a single rural county in the southeast (Okeechobee). Most of the other clusters with relatively high relative risks (RRs = 1.4–1.7) were in multiple counties in northwest, northeast, southwest, and southern regions. During the 2018–2019 time period, ED visit risks were more geographically dispersed compared to the previous period, particularly in rural counties with RRs ranging from 1.2 to 2.8. The cluster with the highest relative risk (Cluster 5; RR = 2.8) was in a small rural county (Wakulla) in the northwest, while additional counties with high ED visit risks (RRs = 2.1–2.5) were observed in the northeast (*i.e.,* Hamilton, Suwannee, Liberty, *etc*.) and the southeast (Okeechobee).

**Figure 4:**
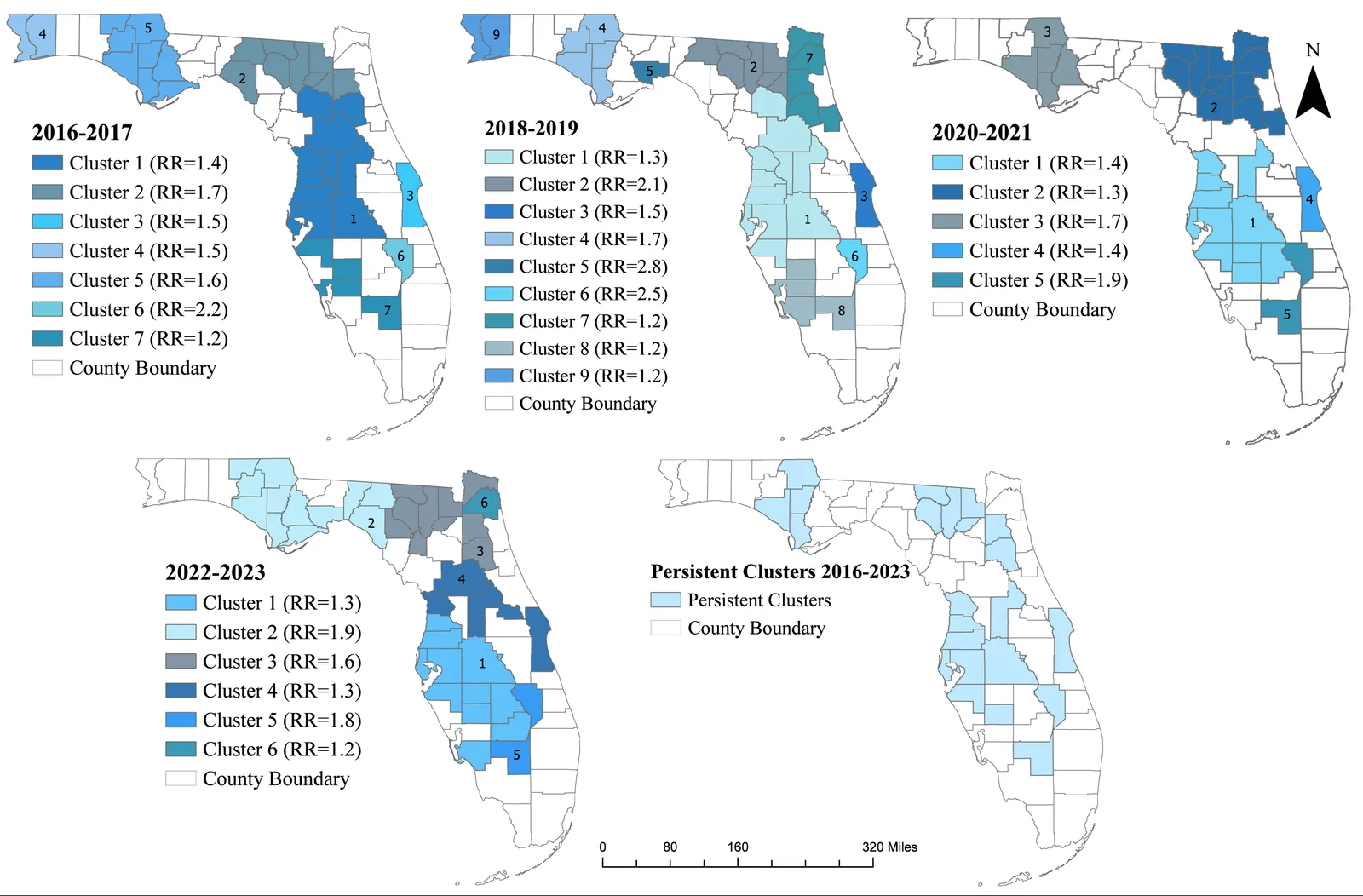
Geographic distribution of spatial clusters of high risk of emergency department visits due to intentional self-harm in Florida, 2016–2023. The map was created by the authors of the study using ArcGISPro software with publicly available cartographic boundary files from US Census Bureau TIGER Geodatabase. The data displayed is from the Florida Department of Health.

**Table 1:** Spatial clusters of high risk of emergency department visits due to intentional self-harm identified in Florida, 2016–2023.

**Period**	**Cluster**	**OC** ^1^	**EC** ^2^	**NC** ^3^	**Population**	**RR** ^4^	***p*-value**
2016–2017	Cluster 1	8,171	6,005	11	4,890,885	1.4	0.001
	Cluster 2	940	541	9	440,307	1.7	0.001
	Cluster 3	1,070	698	1	568,183	1.5	0.001
	Cluster 4	875	585	2	476,702	1.5	0.001
	Cluster 5	618	397	8	323,141	1.6	0.001
	Cluster 6	109	49	1	40,228	2.2	0.001
	Cluster 7	892	751	4	611,517	1.2	0.001
2018–2019	Cluster 1	8,322	6,306	11	5,403,339	1.3	0.001
	Cluster 2	524	254	7	217,631	2.1	0.001
	Cluster 3	1,044	683	1	585,507	1.5	0.001
	Cluster 4	578	332	5	284,808	1.7	0.001
	Cluster 5	106	38	1	32,321	2.8	0.001
	Cluster 6	119	48	1	41,144	2.5	0.001
	Cluster 7	1,917	1,650	5	1,413,742	1.2	0.001
	Cluster 8	1,369	1,163	4	996,170	1.2	0.001
	Cluster 9	692	570	2	488,246	1.2	0.001
2020–2021	Cluster 1	7,698	5,686	11	4,941,826	1.4	0.001
	Cluster 2	2,852	2,244	12	1,949,983	1.3	0.001
	Cluster 3	513	307	5	266,506	1.7	0.001
	Cluster 4	967	692	1	601,573	1.4	0.001
	Cluster 5	171	91	2	79,155	1.9	0.001
2022–2023	Cluster 1	8,133	6,258	11	5,386,501	1.3	0.001
	Cluster 2	923	478	12	411,194	1.9	0.001
	Cluster 3	1,077	687	10	591,274	1.6	0.001
	Cluster 4	2,968	2,371	5	2,040,531	1.3	0.001
	Cluster 5	174	94	2	81,047	1.9	0.001
	Cluster 6	1,347	1,170	1	1,007,189	1.2	0.001

**Notes.**

1 Number of observed cases.

2 Number of expected cases.

3 Number of counties.

4 Relative risk.

During the 2020–2021 time period, there was a temporary decrease in both the number and geographic spread of clusters with high ED visit risks, with RRs ranging between 1.3 and 1.9. Although the overall magnitude of relative risks decreased, two clusters with high ED visit risks (Cluster 3; RRs = 1.7 and Cluster 5; RR = 1.9) persisted, primarily in rural counties located in the northwestern (*i.e.,* Jackson, Washington, Calhoun, and Gulf), the southeast (Okeechobee) and southern (Hendry) region. In 2022–2023, the number of clusters increased to six, with RRs ranging from 1.2 to 1.9. Three major clusters (RRs = 1.3–1.9) covered large portions of northwestern, northeastern, central, southwestern, and southeastern Florida. The number of affected counties increased during this period.

#### Counties in high-risk clusters

The geographic extent of significant spatial clusters expanded over time, reaching its peak during the 2022–2023 time period. Overall, the proportion of Florida counties included in significant clusters of high ED visit risks during the study period ranged from 46.3% (31/67) in 2020–2021 to 61.2% (41/67) in 2022–2023 (Table 2). During the early years (2016–2021), the distribution of clusters with high ED visit risks was not significantly different between rural and urban counties. Specifically, in 2016–2017, the proportion of rural counties that were part of a cluster with high ED visit risk (59.4%; 19/32) was not significantly (*p* = 0.376) different from that in urban areas (48.6%; 17/35). Similarly, in 2018–2019, the proportion of rural counties that were in a cluster with high ED visit risk (56.3%; 18/32) was not significantly (*p* = 0.872) different from that in urban areas (54.3%; 19/35). Finally, in 2020–2021, the proportion of rural counties that were part of a cluster with high ED visit risk (53.1%; 17/32) was not significantly (*p* = 0.282) different from that in urban areas (40.0%; 14/35). However, during the 2022–2023 period, significantly (*p* = 0.001) more rural (81.3%; 26/32) than urban (42.9%; 15/35) counties were included in clusters with high ED visit risks. These findings show that although both rural and urban counties were part of clusters with high ED visit risks during the early years of the study, a notable shift of these clusters with high ED visit risks were more in rural than urban counties later during the study period (2022–2023).

**Table 2:** Rural-urban disparities in county level emergency department visits due to intentional self-harm.

**Clusters**	**Florida (*n* = 67)**			**Rural Counties(*n* = 32)**			**Urban Counties (*n* = 35)**			***P*-value** ^3^
	**No** ^1^	**%**	**95% CI** ^2^	**No** ^1^	**%**	**95% CI** ^2^	**No** ^1^	**%**	**95% CI** ^2^	
2016–2017	36	53.7	41.1–66.0	19	59.4	40.6–76.3	17	48.6	31.4–66.0	0.376
2018–2019	37	55.2	42.6–67.4	18	56.3	37.7–73.6	19	54.3	36.6–71.2	0.872
2020–2021	31	46.3	40.0–58.9	17	53.1	34.7–70.9	14	40.0	23.9–57.9	0.282
2022–2023	41	61.2	48.5–72.9	26	81.3	63.6–92.8	15	42.9	26.3–60.6	0.001
Persistent clusters	23	34.3	23.2–46.9	12	37.5	21.1–56.3	11	31.4	16.9–49.3	0.601

**Notes.**

1 Number of counties within clusters.

2 Binomial exact confidence interval.

3 *P*-value of two sample test comparing proportions of counties in high-risk clusters in rural *vs* urban areas.

Furthermore, over the study period, 34.3% (23/67) of the counties were persistently part of clusters with high ED visit risks. The proportion of rural counties that were persistently in a cluster of high ED visit risks (37.5%; 12/32) was not significantly (*p* = 0.601) different from that in urban areas (31.4%; 11/35). The rural counties that were persistently in spatial clusters with high ED visit risks included: Hamilton, Calhoun, Columbia, Baker, Suwannee, Gulf, Union, Hendry, Okeechobee, DeSoto, Putnam, and Jackson. The urban counties included Bay, Clay, Lake, Citrus, Pinellas, Manatee, Brevard, Hernando, Pasco, Polk, and Hillsborough (Figs. 1 and 4).

#### Population in high-risk clusters

The percentage of rural population living in counties with high ED visit risks increased from 57.2% in 2016–2017 to 71.7% in 2022–2023, compared to 35% in 2016–2017 to 41.8% in 2022–2023 among urban populations (Table 3). A significantly (*p* < 0.0001) higher proportion of the rural compared to urban population lived in counties that were part of clusters with high ED visit risks. Specifically, in 2016–2017, the percentage of rural population living in counties that were part of a cluster with high ED visit risk (57.2%) was significantly (*p* < 0.0001) higher than that in urban counties (35.0%). The disparity widened in 2018-2019 where the percentage of rural population living in counties that were part of clusters with high ED visit risks (63.9%) was significantly (*p* < 0.0001) higher than that in urban counties (44.2%). The gap narrowed slightly during 2020–2021 where significantly (*p* < 0.0001) more rural (59.2%) than urban (35.4%) population resided in counties that were part of clusters with high ED visit risks. The disparity widened again in 2022-2023 where the percentage of rural population living in counties that were in clusters with high ED visit risks (71.9%) was significantly (*p* < 0.0001) higher than that in urban counties (41.8%). Moreover, 6.4 million residents lived in counties that were persistently in clusters with high ED visit risks during the study period. The percentage of rural population living in counties that were persistently in clusters with high ED visit risks throughout the study period (37.9%) was significantly (*p* < 0.0001) higher than that in urban counties (30.1%). These results show that, overall, proportionately more rural populations lived in counties with high ED visit risks than those in urban counties.

**Table 3:** Rural-urban population distribution in high-risk clusters of ED visits due to intentional self-harm.

**Clusters**	**Florida (*n* = 67)**			**Rural counties (*n* = 32)**			**Urban counties (*n* = 35)**			***P*-Value** ^10^
	**TP** ^1^	**CC** ^2^	**%** ^3^	**RC** ^4^	**RCC** ^5^	**%** ^6^	**UC** ^7^	**UCC** ^8^	**%** ^9^	
2016–2017	20,278,447	7,350,963	36.3	1,128,744	645,132	57.2	19,149,703	6,705,831	35.0	<0.0001
2018–2019	20,901,636	9,462,908	45.3	1,155,021	737,529	63.9	19,746,615	8,725,379	44.2	<0.0001
2020–2021	21,339,762	7,839,043	36.7	1,158,650	685,573	59.2	20,181,112	7,153,470	35.4	<0.0001
2022–2023	21,928,881	9,517,736	43.4	1,189,384	855,070	71.9	20,739,497	8,662,666	41.8	<0.0001
Persistent clusters	21,112,182	6,438,615	30.5	1,157,950	438,439	37.9	19,954,232	6,000,176	30.1	<0.0001

**Notes.**

1 **TP:** Total population of Florida.

2 **CC:** Population living in high-risk cluster counties.

3 **%**Percentage of population living in high-risk cluster counties (CC/TP).

4 **RC:** Population living in rural counties.

5 **RCC**: Population in rural counties that were part of a high-risk cluster.

6 **%** Percentage of rural population living in high-risk cluster counties (RCC/RC).

7 **UC:** Population living in urban counties.

8 **UCC:** Population in urban counties that were part of a high-risk cluster.

9 **%** Percentage of urban population living in high-risk cluster counties (UCC/UC).

10 *P*-value of two sample test comparing proportions of population in high-risk clusters in rural *vs* urban counties.

## Discussion

This study investigated county-level geographic distribution and clusters of intentional self-harm-related emergency department (ED) visit risks in Florida. Although the age-adjusted risk of emergency department visits due to intentional self-harm declined slightly statewide, there is evidence of geographic disparities with high ED visit risks occurring disproportionately in rural areas. The findings are useful for guiding evidence-based health planning and need-based allocation of resources geared towards reducing/eliminating disparities and improving health outcomes.

### Temporal and geographic distribution

The decline in the overall state age-adjusted risk of ED visits due to intentional self-harm observed in this study was consistent with those reported by both Ghaderi et al. (2022) and Law et al. (2022) but contrasts with findings from other studies that reported increases (Arakelyan, Emond & Leyenaar, 2022; Bommersbach, Olfson & Rhee, 2024). The studies by Ghaderi and Law attributed the decreases to voluntary self-isolation and stay-at-home orders during the COVID-19 pandemic (Ghaderi et al., 2022; Law et al., 2022). These may be plausible explanations, at least in part, for the decreases observed in the current study. By contrast, Arakelyan and co-workers attributed the increase reported in their study to the relative scarcity of community mental health services in rural areas, necessitating a dependence on the ED to address self-harm emergencies (Arakelyan, Emond & Leyenaar, 2022).

The observed geographic disparities in ED visit risks due to intentional self-harm with high ED visit risks in rural counties may reflect a growing and well documented structural disparity in the distribution of Florida’s mental healthcare workforce (Wang et al., 2022; Brenda, Whetsell & Graves, 2022; Winton, 2022; Brausch, Whitfield & Clapham, 2023; McCarthy et al., 2023; Health Resources and Services Administration, 2023; Saunders et al., 2025). Rural areas tend to have fewer community-based facilities capable of addressing acute psychological distress, which may contribute to higher ED utilization patterns as a result of intentional self-harm in these areas (Ghorbanzadeh et al., 2020; Das, Singh & Bruckner, 2021; Kelley et al., 2024). This may also be a plausible explanation, at least in part, to the high percentage of ED visits attributable to intentional self-harm observed in rural areas in this study. It has also been reported that, in the US, two-thirds of mental health professional shortage areas are in rural counties (Health Resources and Services Administration, 2023). In the US, as many as 65% of rural counties lack a psychiatrist, 47% lack a psychologist and only 2% of all physicians practice in rural America (Brenda, Whetsell & Graves, 2022; Winton, 2022). In Florida, 77.3% of rural counties have inadequate numbers of mental health professionals to meet population needs (Rural Health Information Hub, 2025). Furthermore, insurance regulations under Health Insurance Portability and Accountability Act (HIPAA) require a linked mental health diagnosis in order for ED care for intentional self-harm to be covered by insurance (Marcus, Bridge & Olfson, 2012; Dania, Kevin & JoAnn, 2021). Unfortunately, limited staffing in the EDs can delay timely assessment and documentation of diagnoses, creating logistical/administrative and financial barriers to care (Dania, Kevin & JoAnn, 2021). Unfortunately, a recent study reported that despite the rise in intentional self-harm across the US, less than 16% of self-harm cases that presented at the ED got an evaluation by a mental health professional (Bommersbach, Olfson & Rhee, 2024). Additionally, emerging evidence suggests that digital health and artificial intelligence (AI) applications could be used to supplement scarce mental health providers, particularly in underserved rural areas (Ali et al., 2025; Liu, Ding & Gan, 2026). Future research should explore the feasibility, acceptability, and clinical effectiveness of these technologies specifically for rural counties with high utilization for intentional self-harm.

The shortage of mental health services in rural areas may also suggest that introduction/expansion of population-level evidence-based crisis systems (community health centers, hotlines, mobile crisis units, and stabilization centers) could reduce reliance on the overburdened EDs and improve continuity of care (Das, Singh & Bruckner, 2021; Winton, 2022; Health Resources and Services Administration, 2023). For example, a recent study across 211 counties in 10 US states found that counties with stronger continuity of mental health care within Community Health Centers experienced fewer emergency department visits for intentional self-harm (Das, Singh & Bruckner, 2021). Rural Florida could benefit from similar mobile crisis and stabilization models to reach rural areas with high ED visit risks that currently have low access to mental health services, thereby reducing preventable emergency department visits for intentional self-harm (Kelley et al., 2024). Florida’s Rural Renaissance Legislation (SB 110) passed in March 2025 in part addresses this challenge by providing critical funding to rural hospitals, supporting mobile behavioral health units, and establishing telemedicine kiosks to provide urgent care services remotely in underserved rural areas (Betta, 2025). This critical funding may have the potential to mitigate the high intentional self-harm risks in rural counties across the northwest, northeast, and southern Florida by improving access to behavioral health care and enabling the delivery of preventive and crisis intervention services in locations with limited self-harm prevention infrastructure. Although these factors were not investigated in this study, they provide context in understanding potential determinants of the observed rural–urban disparities, information that should be used to guide future research and targeted interventions.

### Counties in clusters with high ED visit risks

The observed spatial clusters of high ED visit risks due to intentional self-harm in rural counties were similar to reports from previous studies which attributed this to the high utilization of Florida’s Baker Act system in rural socioeconomically disadvantaged areas (Gibbs, 2023; Florida Department of Children and Families, 2024). The Baker Act provides a legal mechanism for involuntary psychiatric examinations of people deemed to be a danger to themselves (Florida Department of Children and Families, 2024). Of the 173,721 Baker Act examinations in the state during the 2022–2023 fiscal year, 57.4% were due to intentional self-harm (Florida Department of Children and Families, 2024). It is worth noting that the 21 counties that were persistently part of clusters with high ED visit risks due to intentional self-harm in this study also reported high Baker Act examinations during the 2022–2023 fiscal year. Suffice it to say that the majority of the Baker Act examinations are in disadvantaged rural counties that are characterized by socioeconomic deprivation, high rates of mental illness, and limited access to outpatient mental health and crisis services (Maniaci et al., 2019; Gibbs, 2023; Florida Department of Children and Families, 2024). While the Baker Act Examinations were not directly investigated in this study, they provide context for understanding plausible hypotheses/contextual interpretation for the observed disparities. Future studies will need to investigate the potential impacts of the Act on these disparities.

### Population in clusters with high ED visit risks

The finding that there were disproportionately more rural residents living in counties located in clusters with high ED visit risks due to intentional self-harm than urban residents is consistent with reports from previous studies (Hoffmann et al., 2021; Wang et al., 2022). Wang et al. (2022) reported that the US rural population had 1.5 times greater rate for age-adjusted emergency department visits for self-harm than urban populations. Additionally, rural–urban comparisons in the US showed that rural youth populations had higher ED visit risks for intentional self-harm than their urban counterparts (Hoffmann et al., 2021). This supports the current study’s finding that rural populations are disproportionately represented among those at high risk of intentional self-harm ED visits.

## Strengths and limitations

A key strength of this study is the use of rigorous spatial epidemiological techniques to identify high-risk clusters and disparities in ED visits due to intentional self-harm. However, this study is not without limitations. The study focuses on disparities in ED visit risks and therefore includes only intentional self-harm cases that result in ED visits, thereby excluding those that either do not seek care or are unable to access ED care. Self-harm is widely recognized as under-reported in healthcare-based data, as many instances are managed outside formal medical settings or go untreated due to various barriers. Therefore, the patterns observed in this study may disproportionately represent populations with greater access to emergency services. Since the outcome is visit-based and not person-based, multiple visits by the same person were treated as separate events. Moreover, estimated ED visit risks from counties with small population may be unstable and should be interpreted with caution. Additionally, cluster detection results may be sensitive to the choice of spatial scan parameters, including the maximum scanning window size and relative risk threshold. Although these parameters were determined *a priori* based on methodological considerations, alternative specifications could potentially yield different results. Rural–urban comparisons were based on county-level observations that may exhibit spatial autocorrelation and temporal dependence, thus violating independence assumptions of standard statistical tests. As such, reported *p*-values should be considered descriptive indicators of differences. This is an ecological study based on county-level data and so inference cannot be made at the individual level. For instance, associations observed between rurality and clusters of high ED visit risks due to intentional self-harm do not necessarily imply rural individuals are at high risk ED visit resulting from intentional self-harm, only that rural areas have elevated intentional self-harm ED visit risks. These limitations notwithstanding, the findings are important for guiding future research, evidence-based health planning, and need-based allocation of resources geared towards reducing/eliminating disparities and improving health outcomes.

## Conclusion

The risk of emergency department visits due to intentional self-harm in Florida declined slightly over the eight-year period of this study. However, there is evidence of geographic disparities with high ED visit risks due to intentional self-harm occurring disproportionately in rural areas. These findings indicate that rural areas may represent important geographical areas for further investigation. Future research should investigate the identified high-risk counties, determine the predictors of the identified disparities, and assess if targeted intervention efforts may reduce the observed disparities.

## Supplemental Information

10.7717/peerj.21963/supp-1 Supplemental Information 1 Study data showing all variables used in the analysis

10.7717/peerj.21963/supp-2 Supplemental Information 2 STROBE Checklist

## Additional Information and Declarations

### Data Availability

The following information was supplied regarding data availability: The raw data are available in the Supplemental Files.
