# Supplementary material for: Geographical disparities in risks of emergency department visits due to intentional self-harm in Florida: a retrospective ecological study
Source: PeerJ. 2026 Nov 12;14:e21963. doi: 10.7717/peerj.21963 (PMC13770425; doi:10.7717/peerj.21963)
Supplement: Supplementary file 1 [file peerj-14-21963-s002.doc]

STROBE Statement—Checklist of items that should be included in reports of ***cross-sectional studies***

|  | | Item No | Recommendation |
| --- | --- | --- | --- |
| **Title and abstract** | | 1 | (*a*) Indicate the study’s design with a commonly used term in the title or the abstract  **Study’s design has been indicated in the title: Lines 1-2** |
|  |  |  | (*b*) Provide in the abstract an informative and balanced summary of what was done and what was found  **Abstract (Background, methods, results, conclusion): Lines 22-51** |
| Introduction | | | |
| Background/rationale | | 2 | Explain the scientific background and rationale for the investigation being reported  **Introduction, paragraphs 1-4: Lines 64-98** |
| Objectives | | 3 | State specific objectives, including any prespecified hypotheses  **End of introduction – paragraph 4: Lines 97-98** |
| Methods | | | |
| Study design | | 4 | Present key elements of study design early in the paper  **Materials and methods (Ethics review, study area): Lines 99-124** |
| Setting | | 5 | Describe the setting, locations, and relevant dates, including periods of recruitment, exposure, follow-up, and data collection  **Materials and methods (study area, data sources): Lines 107-141** |
| Participants | | 6 | (*a*) Give the eligibility criteria, and the sources and methods of selection of participants  **This is a retrospective study using publicly available aggregated data. Details of data are available in Lines 125-141** |
| Variables | | 7 | Clearly define all outcomes, exposures, predictors, potential confounders, and effect modifiers. Give diagnostic criteria, if applicable  **Materials and methods (Data sources): Lines 125-141** |
| Data sources/ measurement | | 8* | For each variable of interest, give sources of data and details of methods of assessment (measurement). Describe comparability of assessment methods if there is more than one group  **Materials and methods (Data sources, calculation of age-standardized ED visit risks and cartographic displays, cluster detection and identification): Lines 125-174)** |
| Bias | | 9 | Describe any efforts to address potential sources of bias  **Materials and methods (calculation of age-standardized ED visit risks and cartographic displays, cluster detection and identification): Lines 143-174**  **Discussion (Strengths and limitations): Lines 327-332** |
| Study size | | 10 | Explain how the study size was arrived at  **Not applicable as this was a retrospective ecological study using secondary publicly available and aggregated data** |
| Quantitative variables | | 11 | Explain how quantitative variables were handled in the analyses. If applicable, describe which groupings were chosen and why  **Materials and methods (Calculation of age-standardized ED visit risks and cartographic displays, cluster detection and identification): Lines 143-174** |
| Statistical methods | 12 | (*a*) Describe all statistical methods, including those used to control for confounding  **Materials and methods (Calculation of age-standardized ED visit risks and cartographic displays, cluster detection and identification): Lines 143-174** | |
|  |  | (*b*) Describe any methods used to examine subgroups and interactions  **Materials and methods (Cluster detection and identification): Lines 156-174** | |
|  |  | (*c*) Explain how missing data were addressed  **Materials and methods (Data sources). No missing data: Lines 140-141** | |
|  |  | (*d*) If applicable, describe analytical methods taking account of sampling strategy  **Not applicable** | |
|  |  | (*e*) Describe any sensitivity analyses  **Not Applicable** | |
| Results | | | |
| Participants | 13* | (*a*) Report numbers of individuals at each stage of study—eg numbers potentially eligible, examined for eligibility, confirmed eligible, included in the study, completing follow-up, and analysed  **Not applicable – no individuals because county-level aggregated data were used** | |
|  |  | (b) Give reasons for non-participation at each stage  **Not applicable – no individuals because county-level aggregated data were used** | |
|  |  | (c) Consider use of a flow diagram  **Not applicable – no individuals because county-level aggregated data were used** | |
| Descriptive data | 14* | (*a*) Give characteristics of study participants (eg demographic, clinical, social) and information on exposures and potential confounders  **Materials and methods (Study area, data Sources, Calculation of age-standardized ED visit risks and cartographic displays): Lines 107-154** | |
|  |  | (b) Indicate number of participants with missing data for each variable of interest  **No missing data. Stated in lines 140-141** | |
| Outcome data | 15* | Report numbers of outcome events or summary measures  **Results (Temporal and geographical distribution of ED visit risks, high-risk spatial clusters): Lines 176-253** | |
| Main results | 16 | (*a*) Give unadjusted estimates and, if applicable, confounder-adjusted estimates and their precision (eg, 95% confidence interval). Make clear which confounders were adjusted for and why they were included  **Figure 2: Age-adjusted ED visit risk; Table 2: Clusters** | |
|  |  | (*b*) Report category boundaries when continuous variables were categorized  **Results (Temporal and geographical distribution of ED visit risks – Jenks classification scheme) Lines 177-192; Figure 2** | |
|  |  | (*c*) If relevant, consider translating estimates of relative risk into absolute risk for a meaningful time period  **Not applicable** | |
| Other analyses | 17 | Report other analyses done—eg analyses of subgroups and interactions, and sensitivity analyses  **Results (High risk spatial clusters: Urban-rural comparisons of counties, relative risks and populations): Lines 193-253.** | |
| Discussion | | | |
| Key results | 18 | Summarise key results with reference to study objectives  **Discussion: Opening paragraph: Lines 255-260** | |
| Limitations | 19 | Discuss limitations of the study, taking into account sources of potential bias or imprecision. Discuss both direction and magnitude of any potential bias  **Strengths and limitations section: Lines 327-332** | |
| Interpretation | 20 | Give a cautious overall interpretation of results considering objectives, limitations, multiplicity of analyses, results from similar studies, and other relevant evidence  **Discussion paragraphs 1-5 and conclusions: Lines 255-339** | |
| Generalisability | 21 | Discuss the generalisability (external validity) of the study results  **Discussion (rural-urban implications) and conclusions** | |
| Other information | | | |
| Funding | 22 | Give the source of funding and the role of the funders for the present study and, if applicable, for the original study on which the present article is based  **Funding section: Lines 349-350** | |

*Give information separately for exposed and unexposed groups.

**Note:** An Explanation and Elaboration article discusses each checklist item and gives methodological background and published examples of transparent reporting. The STROBE checklist is best used in conjunction with this article (freely available on the Web sites of PLoS Medicine at http://www.plosmedicine.org/, Annals of Internal Medicine at http://www.annals.org/, and Epidemiology at http://www.epidem.com/). Information on the STROBE Initiative is available at www.strobe-statement.org.
